# The effectiveness of implementation in Indigenous Australian healthcare: an overview of literature reviews

**DOI:** 10.1186/s12939-016-0337-5

**Published:** 2016-03-10

**Authors:** Janya McCalman, Roxanne Bainbridge, Nikki Percival, Komla Tsey

**Affiliations:** School of Human Health and Social Sciences, CQUniversity, cnr Abbott and Spence St, Cairns, QLD 4870 Australia; The Cairns Institute, James Cook University, PO Box 6811, Cairns, QLD 4870 Australia; Menzies School of Health Research, PO Box 10639, Adelaide Street, Brisbane, QLD 4000 Australia; College of Arts, Society and Education, James Cook University, PO Box 6811, Cairns, QLD 4870 Australia

**Keywords:** Aboriginal and Torres Strait Islander, Indigenous Australian, Health services, Health programs, Review, Implementation, PARiHS framework, Community control, Health equity

## Abstract

**Background:**

Effective implementation can maximise the beneficial impacts of health services. It is therefore important to review implementation in the context of Indigenous populations, who suffer some of the greatest disadvantage within developed countries. This paper analyses Aboriginal and Torres Strait Islander (hereafter Indigenous) Australian health implementation reviews to examine the research question: What is the effectiveness of implementation, as reported in the Indigenous Australian health implementation literature?

**Methods:**

Eight databases were systematically searched to find reviews of Indigenous Australian health services and/or programs where implementation was the focus. Search terms included Aborigin* OR Indigen* OR Torres AND health AND service OR program* OR intervention AND implementation (or like terms) AND Australia AND review. Review findings were analysed through the lens of the PARiHS framework which theorises that successful implementation occurs through the interplay of evidence, context and facilitation. The review followed Cochrane methods but was not registered.

**Results:**

Six reviews were found; these encompassed 107 studies that considered health service/program implementation. Included studies described many health services implemented across Australia as not underpinned by rigorous impact evaluation; nevertheless implementers tended to prefer evidence-based interventions. Effective implementation was supported by clearly defined management systems, employment of Indigenous health workers as leaders, community control, partnerships, tailoring for diverse places and settings; and active facilitation methods. Short-term funding meant most studies focused on implementation in one site through pilot initiatives. Only two mentioned cost effectiveness. Indigenous Australian studies incorporated two elements not included in the PARiHS reference guide: the value of community control and equity of service provision across sites.

**Conclusions:**

Comparison of the Indigenous Australian review findings against the PARiHS reference guide elements suggested a fledgling but growing state of Indigenous implementation research, and considerable scope to improve the effectiveness of implementation. Further research is required to explore Indigenous people’s understandings of what is important in healthcare implementation; particularly in relation to the value of community control and equity issues.

## Background

A previous study by the lead author of this paper claimed that there has been little attention in the implementation science literature paid to researching the implementation of health services and programs targeting Indigenous people globally [1]. This claim was based on a prior systematic search of the Indigenous Australian transfer literature which produced only 14 studies [2] as well as a quick scoping search for global Indigenous health implementation literature in *Implementation Science* which resulted in only six papers. Two years on, the likelihood that effective implementation can maximise the beneficial impacts and cost effectiveness of health services or programs, means that it is important to review whether there has been an improvement in the scope of the health implementation literature targeting Indigenous population groups, who suffer some of the greatest disadvantage within developed countries [2–4]. The term implementation is used to refer to the processes by which a service or program is assimilated into use within an organisation [5].

The limited scope of Indigenous implementation literature in 2013 contrasts with the burgeoning nature of implementation science literature globally. For example, the systematic review of the global diffusion of health innovations literature by Greenhalgh et al. [6] appraised a hefty 1000 relevant full text papers and book chapters spanning the 40 years since Rogers [7] seminal book, *Diffusion of innovations*. Yet an update of the Greenhalgh et al. [6] review using similar search terms for just 8 years (2003–2011) identified more than double that rate - a further 404 publications that identified theoretical implementation models or empirical studies [8]. The scope of conceptual understandings had also expanded – evidenced by the appearance of new concepts and terms since 2002—these included scaling, cross-organisational linkages and clusters, knowledge exchange, knowledge translation and social and professional knowledge networks.

Implementation research in health is growing because of its contribution to understanding how the evidence to practice gap in healthcare can be reduced and the benefits of health services and programs maximised through their effective implementation within diverse contexts [4]. However, a consistent theme in the literature is that reducing the evidence into practice gap is difficult to achieve [4]. The complexity of implementation accounts for part of this difficulty. Thus it is also important to understand the elements that comprise effective implementation, and whether and how these elements are described in the available Indigenous studies.

In Australia, the importance of understanding health implementation in the Indigenous context was recognised by the Lowitja Institute, Australia’s national institute for Aboriginal and Torres Strait Islander health research. In 2011, the Lowitja Institute held collaborative workshops for researchers and community members to inform the improvement of knowledge and understanding about the uptake and implementation of Indigenous health promotion approaches. A project was funded to systematically examine the characteristics, implementation and effects of Indigenous health promotion tools [9] and to develop an Indigenous health promotion tool implementation model [10]. In a related Lowitja Institute project, Brands [11] reviewed the international implementation science literature to determine its relevance to guiding improvements in the implementation of Indigenous Australian health services and programs.

Systematic reviews (or overviews) of reviews provide a method for summarising or scoping the overall evidence from more than one systematic review, and encompassing the results from a combination of populations, interventions, conditions, contexts and outcomes [12]. Reviews of reviews bring together the evidence in one place by synthesising or comparing the findings of related reviews to provide policy makers and practitioner with the evidence they need to improve implementation, taking account of variable quality and scope. This paper builds on the work of the aforementioned Lowitja projects by reviewing the reviews of the Indigenous Australian health implementation literature to determine the scope of the literature and “to make sense of [the] complexity [of implementation], and the elements that require attention if implementation is more likely to be successful” ([13], p. 3).

We applied Kitson et al.’s [14] theoretical model ‘Promoting Action on Research Implementation in Health Services’ (PARiHS) framework in mapping the elements which could provide explanations of the processes by which Indigenous health implementation occurs which could guide improvements in the acceptability, adoption, appropriateness, feasibility, replication, implementation cost, spread and/or sustainability of implemented health services or programs. Brands [11] identified the PARiHS framework as a particularly accessible and flexible international implementation framework that could be usefully applied to Indigenous Australian health services and programs.

The PARiHS framework is based on the theory that successful implementation of evidence into practice is determined by a planned facilitated process involving interplay between three elements: 1) the level and nature of the *evidence* for a health service or program proposed for adoption; 2) the *context* or environment into which the evidence is to be placed; and 3) the *facilitation* or method of implementation [13, 14]. Evidence is defined as the form of knowledge that is made implementable through services and programs, and includes knowledge from research, clinical expertise, and/or local knowledge from clients [13]. Context refers to the environment or setting in which the proposed service or program is to be implemented (including the broad macro, organisational and individual factors) [14, 15]; and the readiness for implementation [13]. Facilitation refers to the process by which change managers help individuals and teams to understand what they need to change and how they need to change it in order to apply evidence to practice [13–15]. Successful implementation encompasses three components: 1) an implementation plan and its realisation; 2) an evidence based practice innovation uptake (i.e., uptake of a clinical intervention and/or delivery system interventions); and 3) the achievement of patient and organisational outcomes [13]. Whilst development of the PARiHS framework was based on observation of implementation practice and testing in four empirical case studies, it has since been further developed and applied in diverse situations [16].

International theoretical understandings of implementation may not adequately explain the implementation of health services and programs to Indigenous population groups within developed countries. However, the PARiHS framework was used in preference to Indigenous theoretical models because there are few theoretical conceptualisations of implementation processes within Indigenous Australian health settings, and those that exist are program-specific [1, 17]. Through applying PARiHS, we also attempted to determine the relevance and utility of this framework for understanding implementation in the Indigenous Australian health context. Hence, the three underexplored research questions were: 1) What is the scope of the Indigenous Australian health implementation literature; 2) What is the level and nature of the evidence that underpins implementation, the contexts into which the evidence is placed, and the methods for facilitating implementation; and 3) Is the PARiHS framework useful for understanding implementation in the Indigenous Australian health context? Thus, this systematic review of systematic reviews was conducted to appraise, summarise and bring together the findings of Indigenous Australian health implementation reviews and compare and contrast these with those elements outlined in the international PARiHS framework as being critical to successful implementation.

## Methods

We did not develop a written review protocol, although the focus and methods of the systematic search were established prior to beginning work. The methods were based on Cochrane guidelines and prior reviews by the author team [2, 9, 18–21]. Reviews of the Indigenous Australian healthcare implementation literature were identified and classified using a process that was consistent with PRISMA-P (Preferred Reporting Item for Systematic Review and Meta-analysis) guidelines [22].

### Searches

**Fig. 1:**
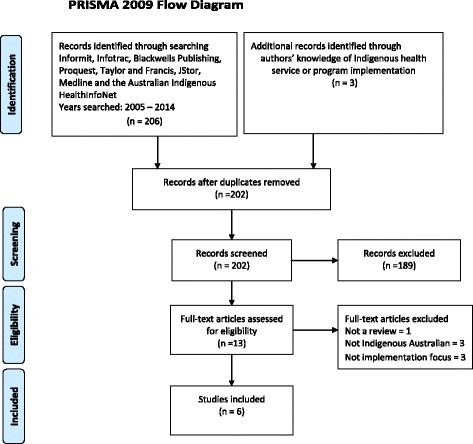
PRISMA 2009 flow diagram

The search strategy is summarised in Fig. 1. First, eight electronic databases were searched: Informit, Infotrac, Blackwells Publishing, Proquest, Taylor and Francis, JStor, Medline and the Australian Indigenous HealthInfoNet. The following terms were searched in either the title or abstract, article or MESH heading of peer reviewed papers: Aborigin* OR Indigen* OR Torres AND health AND service OR program* OR intervention AND implementation (or like terms listed below) AND Australia AND review. In consideration of the broad and expanding international implementation literature, search terms related to implementation included: dissemination, extension, transfer, translation, adaptation, uptake, utilisation, spread and scaling.

Separate searches were performed for each database using database specific subject headings and keywords. The combined searches of the databases produced references that were imported into a bibliographic citation management software EndNote X7. The authors of this study also drew on their knowledge of Indigenous healthcare implementation literature to identify papers.

### Inclusion criteria

To capture evidence of Indigenous Australian health implementation, studies were included if they if they were reviews (systematic, rapid, narrative or other); focussed on health services and/or programs for Indigenous Australians; implementation was the focus of the review; and studies were published in the English language between 2005 and 2014 (inclusive) in the peer review literature. The search was limited to peer reviewed studies as a marker for quality reviews. Given that implementation is a relatively new field of study, a decade of literature was considered sufficient for analysing the majority of Indigenous health implementation studies and was feasible within the scope of this project.

### Review process

Electronic database searching was completed on June 26th, 2015 and yielded 206 citations. An additional three reviews were added based on the authors’ knowledge. After duplicates were removed, 202 citations were screened by one author (JM) to remove articles that were clearly not relevant to the review based on the title, abstracts, journals and keywords of the articles. This screening resulted in 189 citations being excluded from the review.

For the studies considered to meet the review’s eligibility criteria, copies of full text articles were obtained (*n* = 13). Two reviewers (JM, RB) independently assessed the 13 articles with 77 % agreement. Discrepancies were resolved by discussion. Seven studies were excluded from the review for the following reasons: Not a review of the literature (*n* = 1), not Indigenous Australian (*n* = 3), not focussed on implementation (*n* = 3). Ultimately, six studies were included in the analysis, including two of the reviews that had been added based on the authors’ knowledge [2, 23].

### Quality of studies

The quality appraisal methods used by the authors of the included reviews were taken at face value and are reported in Table 1.

**Table 1:** Reviews of the Indigenous Australian health literature that focus on implementation

Author and year	Population	Intervention focus	Review type	No. Indigenous Australian studies	Quality measure
Clifford et al. [23]	Indigenous Australians	The dissemination of Indigenous smoking, nutrition, alcohol and physical activity interventions	Systematic search, 1990–2007	11	Not stated
Gibson et al. [26]	Indigenous people with a chronic disease, their family or community members, PHC providers and policy and decision makers working in Indigenous health	Primary health care interventions for Indigenous people with chronic diseases	Systematic review of published and unpublished literature in English, 1998–2013	18	Joanna Briggs Institute System for the Unified Management, Assessment and Review of Information instrument (JBISUMARI)
Gray et al. [30]	Indigenous Australians undergoing or needing alcohol treatment, and their families, and alcohol treatment providers.	Alcohol treatment among Indigenous Australians: A thematic review of five research projects	A thematic review of papers related to five research projects, 2007–2010	5	Not stated
McCalman et al. [9]	Indigenous individuals, families, organisations and communities	Indigenous health promotion tools	A systematic literature search, 2005–2014	22/65 studies that described or evaluated Indigenous Australian health promotion tools considered their implementation	Quantitative studies: Dictionary for Effective Public Health Practice Project (EPHPP); Qualitative studies: Critical Appraisal Skills Program (CASP).
McCalman et al. [2]	Indigenous Australians	The transfer of Indigenous health services and programs	Systematic search, 1992–2011	20 studies (including 7 protocols) and 1 review [23]	Peer review or not; used an experimental design or not
Ridani et al. [29]	Australian Indigenous Communities	Suicide Prevention past and present programs	Systematic search of grey literature through databases and websites, 1998–2012	46 papers describing 67 suicide prevention programs, of which 59 described implementation	Not stated

### Classification of studies

Aligned with the PARiHS framework, studies were categorised by the first author and year, service or program name, evidence that informed the service or program, organisational context and method for facilitating implementation.

## Results

### Reviews and included studies

We identified six systematic searches of the Indigenous Australian health literature that focussed on implementation and fit the eligibility criteria (Table 1).

These six reviews incorporated 107 Indigenous Australian healthcare implementation studies (after removing duplicates) which included evaluations and descriptions of the implementation of services and programs to address diverse health conditions, services and systems. Only 11 of the studies were included in more than one review, with two studies appearing in three reviews. The six reviews focussed on chronic disease; smoking, nutrition, alcohol and physical activity (SNAP) interventions, suicide prevention, alcohol and other drug treatment and health promotion tools. The reviews utilised differing terms to describe similar concepts of implementation. In addition to the term “implementation” which is defined above, the related terms utilised were: 1) dissemination (the extent of uptake of evidence-based interventions by health-care providers) [24]; 2) transfer the process and practice by which an initiative is made available and accessible to a new setting through interactive engagement between organisational representatives and participants; 3) uptake (the decisions made, often by multiple agents, to make full use of an initiative as the best course of action available) [6]; and spread (the idea that a program expands to increase the number of people served) [25].

Only three of the reviews employed a measure of study quality, with the quality of studies generally found to be weak or moderate. For example, Gibson et al. [26] used the JBI SUMMARI tool and found the quality of qualitative and quantitative studies overall to be moderate, mainly due to insufficient description of the methodological approach. McCalman et al. [9] assessed the quality of quantitative evaluation studies using the Effective Public Health Practice Project (EPHPP) tool and qualitative studies using the Critical Appraisal Skills Program (CASP) tool, and found it to be strong for only five/74 (7 %) studies. McCalman et al. [2] rated quality using peer review and study design as quality measures and found that none of the evaluation studies were based on experimental research designs. These findings of generally poor methodological quality were not surprising since Indigenous health research globally and locally has been predominantly descriptive [27]. Reflecting the logistical, ethical and methodological challenges associated with conducting rigorous intervention and implementation research in Indigenous health-care settings, few studies have met rigorous methodological criteria [27, 28].

### Synthesis of findings

A synthesis of the findings from the Indigenous Australian health implementation reviews is provided here. We analysed the findings according to the research questions.

### What is the level and nature of the evidence that underpins implementation?

Reviews stated that in many cases, the new services and programs that are constantly being introduced into Indigenous primary healthcare services had not been previously systematically evaluated to determine their effectiveness. For example, McCalman et al. [9] found that the impact of only 15 % of Indigenous health promotion tools implemented had been evaluated and McCalman et al. [2] found that only 31 % of 119 studies that reported transfer of an Indigenous health service or program had evaluated the impact of the service or program. Reviews recommended continuing and improving use of valid and reliable measures and rigorous research designs are required to accurately quantify the effect of implementation strategies [2, 9, 23, 29].

However, there was some evidence from reviews that implementers may have been aware of the research evidence and preferenced the implementation of evidence-based services or programs. For example, Clifford et al. [23] reported that all but one of the 11 studies included in their review explicitly reported using evidence-based resources and/or guidelines. McCalman et al. [2] found that of 37/119 transfer studies (31.1 %) evaluated the impact of the service or program compared to only 16.7 % of impact evaluation studies among the remaining 1192 publications reviewed. Thus, while the evidence for what works in improving Indigenous Australian health has been dubbed “the sorry state of the evidence base” ([28], p. 566), in some cases the available evidence was being accessed to inform the choice of which intervention to implement.

While the state of the research evidence can make it difficult for practitioners to confidently select innovations that will guide improvements in health practice, Gray et al. [30] argued that concerns about the scientific rigour of the research need to be balanced by the overriding importance of Indigenous control of the research process. Indigenous control encompasses the identification of the research topic and building the capacity of Indigenous researchers to disseminate, translate and implement results [30]. When Indigenous people had control, the credibility of the evidence source was enhanced with Indigenous end users. Reviews also profit from Indigenous co-authorship, since the interpretation of study findings from an Indigenous worldview perspective demonstrates respect and increases the likelihood of a converging interpretation of the aims and targets of implementation, and hence of research benefit [31]. Five of the six reviews [2, 9, 23, 26, 30] were identified as being co-authored by Indigenous researchers.

Paramount within Indigenous Australian health implementation reviews were issues related to what constitutes evidence. Reviews described implementation processes as best serving Indigenous populations when they drew from both the available scientific evidence as well as, importantly, the knowledge of local Indigenous people and healthcare workers to inform locally relevant program implementation. McCalman et al. [9] found that 70 % of publications that described or evaluated the development of Indigenous health promotion tools specified that community members were consulted or collaboratively involved in developing or adapting the tool. Gibson et al. [26] iterated the importance of involving communities in the design and implementation of services and programs to ensure that their particular health concerns were addressed. Similarly, Gray et al. [30] considered that optimising and maintaining investment required recognition of cultural difference in both the planning and delivery of alcohol services and programs. They stated (p. 487):

Aboriginal community controlled organisations (ACCOs), their practices and values reflect the groups that established them and which they serve. These cultural elements affect the relationships between Aboriginal and mainstream organisations, implementation of specific interventions within ACCOs, and patient-practitioner relationships… recognition of cultural differences is central to modifications to the AUDIT and ‘Drinkless’ materials by the SSWAHS team; and the clash of cultural values and failure to recognise differences, highlighted in the AADS study, demonstrates how provision of quality care can be undermined.

However, few reviews explicitly considered how Indigenous knowledge (such as the principles underpinning health programs and services) were reflected in program or service implementation. In one exception, in the context of suicide prevention, Ridani et al. [29] exemplified the importance of recognising the divergent interpretations of implementation from Western and Indigenous perspectives. Reflecting the importance of engagement with Indigenous knowledges in designing services and programs and in interpreting the outcomes of their implementation, the authors found that while non-Indigenous run programs were individualistic and treatment oriented, Indigenous run programs were more community-focussed and holistic. Additionally, Gray et al. [30] noted conflicts between varying knowledge sources, including: “divergent views regarding staff skills and competencies, including the relative importance of clinical and cultural competencies.” Thus, although reviews recognised the role of evidence from quality research, clinical experience and local knowledge to tailor services or programs appropriately for the context, this was reported inconsistently and evidence was likely to have been inconsistently embedded in service or program implementation.

### What are the contexts into which the evidence is placed and how does implementation work within diverse health contexts?

Health services and programs are implemented across widely varying geographical, historical, social and cultural contexts. This diversity means that implementation efforts need to be tailored to local contexts and the effectiveness of implementation can vary considerably, even for the same service or program. At a local level, Indigenous Australian reviews documented the implementation of health services and programs by government departments, peak or “hub” provider organisations, primary healthcare organisations, partnerships and networks, training organisations, schools, regional health organisations, men’s groups, sports clubs, general practice and non-government organisations [9]. Within organisations, implementation was facilitated by primary healthcare workers, health specialists, Indigenous community members, health promotion officers, policy makers, community/welfare workers, specialist alcohol and other drug, tobacco, mental health, and sexual health workers, and Indigenous and non-Indigenous academics [9]. As employees, these individual change agents had acted for the organisations’ interests but maintained some discretion to interact, advocate and negotiate for how implementation was facilitated.

Some community and organisational contexts were more conducive to the successful implementation of health services and programs than others. Within organisational settings, studies identified the benefits of support from management. Clearly defined management structures and procedures were identified as important in the provision of Indigenous alcohol and other drug services; Gray et al. [30] highlighted the need to formalise processes and commitments within organisational policy and procedures. Community control was mentioned in several reviews as facilitating implementation of health services/programs to Indigenous community members. Mechanisms by which this occurred were by enabling community engagement with service or program design [26], promoting cultural activities and other community-focussed interventions that fostered a sense of community connectedness and pride [29], optimising spread by providing services targeted to Indigenous Australians [23]; and committing to program longevity [26, 29].

A key facilitator of implementation was the employment of local Indigenous health workers. Implementation was facilitated through their roles as cultural mentors to non-Indigenous staff and ability to provide a culturally safe service [26]. Gibson et al. found that the participation of Indigenous health workers in all levels of decision making relating to chronic disease management was particularly important for implementing appropriate and effective services to Indigenous patients and their families.

Barriers to implementation included recruiting qualified staff, high staff turnover and the use of temporary staff. For example, Gray et al. [30] reported on a workplace survey to assess the needs for staff training within an alcohol and other drug service, and development of a training program. However, high staff turnover precluded completion of the outcome evaluation of the training program within the project time frame [30]. Reviews emphasised the need for appropriate staff development, including time to attend training and a backfill of staff; a dedicated coordinator and/or facilitator role [9] and change management and communications plans.

A commitment to partnerships, collaboration and networking was also important [9, 23, 26, 30]. Gray et al. [30] reported on a partnership for the provision of counselling, withdrawal management and residential rehabilitation for Indigenous clients. The authors described the partnership as “fraught with tension” as a result of a range of structural, historical, cultural and personal factors—compounded by client complexity and the “paternalism of the funding agency” (p. 485). Despite these challenges, most staff interviewed considered the potential for partnerships and the need for such partnerships to be voluntary, equitable, accountable and based on trust. Partnerships between Indigenous practitioners and researchers were seen to be a positive outcome of the implementation process [9, 30]. A collegiate approach encompassing the sharing of ideas in the development of research proposals and projects, training, and the funding of projects was valued and led to significant improvements in research design, process and outcomes [30].

All reviews also cited macro political, social and economic factors, such as government policies, funding amounts and duration, and the economic and social determinants of Indigenous health, that influenced the implementation of health services and programs [2, 9, 26, 29, 30]. In particular, the provision of adequate and enduring funding for Indigenous health service and program implementation was required for health infrastructure and recruiting, training and support for additional members of the health workforce, as well as to continue the delivery of services or programs. For example, Gibson et al. [26] stated:

Indigenous-specific services often tended to rely on a multitude of short-term government funding arrangements which threaten their sustainability and result in overwhelming reporting requirements. Funding arrangements between Indigenous community-controlled health services and governments tend to be more complex than those between governments and general practice or tiers of government in Australia and elsewhere. One of the key issues to be considered during the design phase is therefore adequate funding for both the implementation and sustainability of an intervention.

Reviews critiqued the short-term nature of funding grants as inefficient; finding that the uncertainty of funding created a barrier to sustained implementation [2, 26, 29, 30]. For example, Gray et al. [30] found that the short-term nature of funding exacerbated problems in alcohol and other drug treatment such as the difficulty of recruiting qualified staff (particularly in rural and remote areas), establishing collaborative relationships and gaining community acceptance. In contrast, modest additional resources to alcohol and other drug projects produced change and enhanced outcomes through increased capacity to deliver services; improved case identification; increased client engagement; improved interagency and community collaboration; and development of more appropriate assessment tools and resources. As these became embedded in service provision, the initial investment continued to have a positive effect and this success led to further funding allocations by government agencies [30].

Studies reported wide variations in implementation between local areas. For example, McCalman et al. [9] highlighted the findings of an included study by Tursan d’Espaignet et al. [32] of significant improvements in birth weight following the introduction of the Strong Women, Strong Babies, Strong Culture Program in one group of Indigenous communities in the Northern Territory with no significant change in the second group. The authors concluded that there was a need to better understand how implementation of the same program differed across the community contexts. This finding implied a need to identify ways to support implementation of the strategies in areas of greatest need; thereby making the implementation of services and programs more effective and equitable. It also suggests that services/programs can not necessarily be directly transferred across contexts; for example, from mainstream general practice to Indigenous health services; between urban, rural and remote settings; or between communities.

### What are the methods for facilitating implementation and what facilitation strategies work?

The methods for facilitating the delivery of Indigenous health services or programs affected the success of the service or program. Drawing from a typology of types of spread identified by Ovretveit [3], McCalman et al. [2] found that the most common type of transfer and implementation of health services and programs reported in studies was through the central development but decentralised implementation of the initiative; this involved community-based participation and adaptation of the intervention (12/21 or 57 % studies). Also found were studies of informal grass roots transfer (5/21 studies). An example was provided by Ridani et al. [29] who reported the lateral sharing of suicide prevention knowledge between communities as a process “vital in empowering communities to help each other” (p. 25). The third type of transfer found was hierarchical top-down implementation (3/21 studies).

Reviews considered that services and programs effective in non- Indigenous communities could not simply be implemented in Indigenous settings without consideration of cultural differences [30]. Local Indigenous knowledge was considered important in tailoring mainstream health services and programs to fit the diverse contexts of Indigenous Australian health. For example, Gray et al. [30] noted that the provision of training and tailored outreach support resulted in modest evidence of improvements in alcohol screening in community controlled health services. However, McCalman et al. [9] reported that the majority of Indigenous health promotion tools were designed for national or state/territory use, with only 12 % developed or tailored for regional or local use.

Reviews cited the critical importance of Indigenous leadership, governance and involvement in all aspects of decision making and responses to issues affecting their lives. For example, Ridani et al. [29] found that suicide prevention programs that were wholly or partly Indigenous owned, employed creative and community-focussed strategies such as art classes, dancing events, theatrical showcases and cultural camps; whereas those not owned or run by Indigenous corporations used workshops as their main mode of delivery. Reviews cited the need for respect for Indigenous peoples’ information and knowledge systems, safe spaces for knowledge exchange, and strengthening of trust, engagement and participation within health and other systems.

Reviews also described how passive implementation strategies, such as the distribution of clinical guidelines or resources, were generally ineffective compared to the use of actively facilitated implementation, which increased readiness for implementation [9, 23, 26]. Active facilitation roles were provided by agents external to the implementation context, such as researchers, and by internal change agents, such as continuous quality improvement managers or health practitioners. Gibson et al. [26] cited the quality of patient/provider partnerships as one of five key factors that enabled or inhibited the implementation of services and programs aimed at improving chronic disease primary health care for Indigenous people. Developing respectful, trusting and safe relationships with providers was particularly important given historical policies and practices that excluded and discriminated against Indigenous people; and achieving this was often time consuming and required effort and understanding on the part of the provider.

In corollary, McCalman et al. [9] highlighted an included study by Hunter et al. [33] that found that distribution of clinical resources alone was not sufficient to ensure use. The evaluation of the National Recommendations for the Clinical Management of Alcohol-Related Problems in Indigenous Primary Care Settings found that although these clinical guidelines were produced through a series of workshops involving some 15 expert clinicians and workers in the area of Indigenous primary care and substance use; their dissemination to doctors and nurses working with Indigenous patients required 74 workshops conducted by an Indigenous and non-Indigenous medical practitioner and worker with expertise in Indigenous alcohol use. Particularly for medical practitioners, appropriate introduction by acknowledged experts not only increased use, but also positively influenced willingness to reflect on their interactions with Indigenous drinkers and to engage with alcohol-related problems using the guidelines as part of primary clinical care. Hence, the credibility of the new guidelines for end users was influenced not only by the use of an evidence-based intervention, but also by the perceived level of expertise and active facilitation of the implementation process.

From their review of dissemination strategies for Indigenous Australian SNAP interventions, Clifford et al. [23] found that half of the studies employed a combination of strategies that was less than optimal. Dissemination strategies included academic detailing (educational outreach or skills training); continuing medical education; reminder systems, and reinforcement contact (structured follow-up contact with practitioners to reinforce education or training). Most studies reported the tertiary prevention of diabetes care, rather than the uptake of secondary preventive interventions such as brief intervention in Indigenous primary health care settings.

### What works in successfully implementing health services and programs for Indigenous Australians?

Several reviews reported a need to plan implementation. For example Gray et al. [30] urged: “to optimise and maintain investment, cultural difference needs to be recognised in both planning and delivery of alcohol interventions”. Yet generally, implementation planning was considered primarily when plans were abandoned due to unexpected contingencies. For example, Gray et al. [30] reported: “staff turnover led to abandonment of a plan to conduct a post-intervention evaluation survey” and “initially, it was planned to conduct the individual projects over a 12-month period. However, all projects exceeded this” (p. 487). The use of formative evaluation to refine implementation plans to account for such unanticipated factors was reported. For example, McCalman et al. [2] found that 28/119 (23.5 %) studies reported only process evaluation measures of reach, satisfaction, quality and how implementation occurred. However, these examples point to a need for improved reporting of implementation planning and refinement.

The reported outcomes of implementation strategies included the uptake of secondary and tertiary preventive interventions, tailored to clinic, patient and health-care provider factors in specific settings, and cost-effectively implemented across multiple Indigenous health-care services and programs [23]. Also reported as positive outcomes were the close networks established between practitioners and researchers [9, 30]. Yet, consistent with perceptions of Indigenous people that research has produced little health benefits [34], the perceptions of Indigenous stakeholders in terms of the usefulness of such changes were not reported.

Gray et al. [30] noted that the transfer and uptake of services and programs encompasses a long-term process. Although all reviews cited the importance of sustaining service or program implementation, most of the included studies focused on singular incidents of implementation through pilot initiatives. Program longevity tended to be linked to community ownership [29]. Ridani et al. [29] concluded:

Piecemeal and ad hoc approaches are unlikely to be effective and near impossible to evaluate. Perhaps the challenge in moving forward will be to determine how we can coordinate intervention efforts so that they not only have an effect, but they also demonstrate it.

The spread of services or programs to other sites was quantified by McCalman et al. [2]. Of 1311 publications which they identified as dealing with Indigenous Australian health services, programs or innovations, 119 (9.1 %) referred to their transfer. Transfer or implementation was the primary focus of 21 (1.6 %) of these studies and was only considered by the remaining 98 (7.5 %) studies. The authors concluded that few studies focus on the process by which transfer or implementation of health services or programs occurred or their effectiveness in the new site.

The cost effectiveness of implementation was mentioned in only two of the six reviews [23, 30]. Based on an audit showing that few Indigenous people were accessing alcohol and other drug services in Sydney’s South West, Gray et al. [30] reported a project to assess the potential role of ‘community-based education and brief intervention’ in reducing harm. The intervention was labour intensive, comprising screening of community group members; interactive education sessions to increase awareness of alcohol-related harms, alcohol guidelines and availability of services; and feeding back screening scores and providing one-to-one brief interventions for those at risk. The authors suggested positive results and potential economies of funding, staffing and training if the approach became a routine element of service provision. Nevertheless, both Gray et al. [30] and Clifford et al. [23] suggested a need for further examination of cost-effectiveness.

## Review limitations

The method used to search electronic databases may not have located all Indigenous Australian health implementation reviews published in the peer reviewed literature from 2005 to 2014. Only a small number of implementation-focussed reviews were found however, given the small number of primary studies, this was not surprising. Limitations in the findings of this analysis include potential bias given the small number of papers found and the first authorship of two of the reviews by the first author of this paper. Gaps in the information provided by the studies include foci on only a limited range of Indigenous health issues (chronic disease, SNAP interventions, suicide prevention, alcohol and other drug treatment and health promotion tools). Systematic reviews of reviews generally create a meta-analysis of the included reviews; however the descriptive foci of the included reviews meant that this was not feasible.

## Discussion

More than ten years ago, Ring and Brown [35] critiqued the slow progress in Indigenous Australian health improvement as due to “lack of commitment to and implementation of already existing policies”. The reviews which provided the basis for this paper suggest that there is a growing body of published research that describes or evaluates the implementation strategies that would reliably result in health improvement, assist in accessing hard-to-reach community members, or provide best value for money. However, the quality of studies is poor or moderate. This paper found value in the PARiHS theoretical model for identifying the broad elements critical to implementing Indigenous Australian health services and programs. Application of the PARiHS framework to the Indigenous Australian healthcare implementation literature identified a range of key policy and management issues where there is scope for improvement.

Table 2 provides a mapping of the findings of Indigenous Australian health implementation reviews to the PARiHS framework elements. Included in that table are the key elements highlighted in the PARiHS reference guide [13]; the comparable findings in the Indigenous Australian implementation reviews; elements that were highlighted in the PARiHS framework but missing from the Indigenous Australian reviews; and elements highlighted in the Indigenous Australian reviews but missing from PARiHS. It should be noted that the missing elements are not necessarily absent in practice; simply that they were not reported in reviews.

**Table 2:** Mapping the findings of Indigenous Australian health implementation reviews to the PARiHS framework elements

	PARiHS – conditions needed for successful implementation	Indigenous Australian implementation – what was included	Indigenous Australian implementation – what was missing	PARiHS – what was missing
1) What is the level and nature of the evidence that underpins implementation	• Research and published guidelines • Clinical experiences and perceptions • Patient experiences, needs, and preferences • Local practice information • Characteristics of the targeted EBP: • Relative advantage • Observability • Compatibility • Complexity • Trialability • Design quality and packaging • Costs	Many health services and programs being implemented were not underpinned by rigorous evaluation of their effects. However it seemed that more evidence-based programs were being implemented than the proportion reported in the literature – i.e., implementers were aware of the evidence and implemented evidence-based programs. Research quality of implementation studies was poor or moderate.	Reviews mentioned clinical and patient experience as a source of evidence, but did not elaborate what local data was available or accessed, nor how this knowledge was embedded in practice.	Reviews highlighted the value of Aboriginal control of the research process.
		Reviews highlighted the value of local Indigenous knowledge in developing and implementing services and programs.		
2) What are the contexts into which the evidence is placed and how does implementation work within diverse health contexts	• Leadership support • Culture • Evaluation capabilities • Receptivity to the targeted innovation/change	Reviews recognised the need for clearly defined management structures and procedures. Commitments to employment of local Indigenous health workers as leaders supported implementation.	The extent to which managers (or other leaders) supported implementation was not made explicit.	To improve the equity and effectiveness of service provision, support for the implementation of services or programs in areas of greatest need was seen to be warranted
		Barriers to implementation were reported, e.g., staff recruitment and retention, staff development, the availability and designation of implementation leaders and absence of implementation or communication plans.	The extent to which key stakeholders collaborate, value open dialogue, support implementation and see it as appropriate to their role was not reported.	
		Short-term funding exacerbated problems in service implementation whilst modest additional resources produced change and enhanced outcomes.	The extent to which targeted sites had resources (expertise and systems) to access baseline and other evaluative data, or evaluated implementation, was not reported.	
		Community control enhanced credibility and enabled community engagement, cultural activities and commitment to service or program longevity.	The extent to which communication channels, formal networks, internal facilitation resources and authority, and the fit of implementation with organisational priorities was not reported.	
		Effective partnerships, and collaboration and networking between government and research agencies, health-care providers and Indigenous primary healthcare services increased the likelihood of implementation success.		
3) What are the methods for facilitating implementation and what facilitation strategies work?	Role of facilitator:	Three key types of facilitation were found: participatory, grass roots and hierarchical. The need for tailoring implementation across sites was recognised. Implementation was facilitated by external and internal change agents, and enhanced through Indigenous leadership, governance and support for implementation.	The type of facilitation role needed for each type of implementation (e.g., external or system level facilitator) was not explicated; nor was the availability of individual facilitators with appropriate attributes, skills and expertise.	The importance of sustaining service or program implementation was reported, however most of the included studies focused on singular incidents of implementation through pilot initiatives. The cost effectiveness of implementation was mentioned in only one review.
	• Purpose, external and/or internal role • Expectations and activities • Skills and attributes of facilitator			
	Other implementation interventions suggested per site diagnostic assessment or relevant sources (e.g., prior research/literature and supplementary theories) and used by the Facilitator and others	Active facilitation worked better than passive dissemination methods. Reviews cited implementation of more than one facilitation strategy, but mix was not optimal.		
	• Related to E • Related to C • Other			
4) What works in successfully implementing health services and programs for Indigenous Australians?	Implementation plan and its realisation	Reviews reported a need to plan implementation, yet planning was considered primarily when plans were abandoned due to unexpected contingencies. Formative evaluation to refine implementation plans was reported.	The perceptions of Indigenous stakeholders in terms of the usefulness of such changes was not reported.	
	Evidence-based practice innovation uptake of clinical interventions and/or delivery system interventions	All reviews cited the importance of sustaining service or program implementation, most of the included studies focused on singular incidents of implementation through pilot initiatives.		
	Patient and organisational outcomes achievement	Few studies focus on the process by which transfer or implementation of health services or programs occurred.		
		The cost effectiveness of implementation was mentioned in only two reviews.		

Comparison of the Indigenous Australian review findings against the elements outlined in the PARiHS reference guide found a high level of consistency. For example, although the implementation of many Indigenous health services and programs was not underpinned by high quality or tailored impact evaluation of their effects, where available, implementers preferred evidence-based programs. Implementation success for Indigenous health services and programs was enhanced by recognition of the value of local Indigenous knowledge, clearly defined management systems, commitments to employing Indigenous health workers as leaders, community control, effective partnerships, tailoring for diverse sites; and active facilitation methods. Reported barriers to implementation included poor staff recruitment and retention, inadequate staff development, the unavailability of designated implementation leaders and absence or inadequacy of implementation or communication plans. All reviews cited the importance of sustaining service or program implementation, yet short-term funding meant that most included studies focused on singular incidents of implementation through pilot initiatives, and only two mentioned cost effectiveness.

PARiHS was not useful for explaining the value of community control and improvement of equity of service provision across sites. However, comparison with the PARiHS framework did identify five elements that were missing from the Indigenous Australian health implementation reviews. These were the extent to which: 1) managers and other stakeholders supported implementation and saw it as appropriate to their roles; 2) internal facilitation resources, authority, skilled facilitators, communication channels and networks were available; 3) implementation fit with organisational priorities; 4) local or other evaluation data was used to inform practice and evaluate implementation; and 5) Indigenous stakeholders perceived the usefulness of changes through implementation.

## Conclusion and implications

There are three key implications of this analysis of Indigenous health implementation reviews. First, to support the implementation of evidence-based interventions, methodologically rigorous evaluations of current services and programs are needed which use outcome measures with demonstrated reliability and validity to quantify the effect of changes in health service delivery. As well, further research is required to explore Indigenous people’s understandings, principles and knowledge of what is important in healthcare implementation; particularly in relation to the value of community control and equity issues. Second, contexts that support sustained health service or program implementation include the provision of adequate and enduring funding. The short-term nature of funding exacerbated problems in service delivery whilst modest additional resources produced change and enhanced outcomes, leading to further funding allocations. To improve the equity and effectiveness of service provision, support for the implementation of services or programs in areas of greatest need is warranted. Finally, implementation could be better supported through enabling Indigenous leadership, governance and involvement in implementation; tailoring services and programs; and active facilitation methods to fit the diverse contexts of Indigenous Australian health settings.

## Abbreviations

PARiHS: Promoting Action on Research Implementation in Health Services
